# Discovery of a second, distinct development pattern of leukemic conversion from paroxysmal nocturnal hemoglobinuria

**DOI:** 10.1007/s12185-025-03923-3

**Published:** 2025-02-06

**Authors:** Junji Tokushige, Kazuki Taoka, Masako Nishikawa, Masahiro Jona, Kumi Nakazaki, Yutaka Yatomi, Mineo Kurokawa

**Affiliations:** 1 Department of Hematology and Oncology, Graduate School of Medicine, The University of Tokyo, 7-3-1 Hongo, Bunkyo-ku, Tokyo, 113-8655 Japan; 2 Department of Clinical Laboratory, The University of Tokyo Hospital, Tokyo, Japan; 3 Department of Cell Therapy and Transplantation Medicine, The University of Tokyo Hospital, Tokyo, Japan

**Keywords:** Paroxysmal nocturnal hemoglobinuria, Leukemic conversion, A second development pattern

## Abstract

The incidence of leukemic conversion during the clinical course of paroxysmal nocturnal hemoglobinuria (PNH) has been reported to be 0.6–2.9%. Such an association is logically linked to the progression of PNH to acute leukemia, especially the M6 subtype of acute myeloid leukemia (AML-M6). In many of these cases (11/26, 42%), leukemic conversion from PNH is associated with development of AML-M6. A literature review including our cases showed that this leukemic conversion from PNH has two distinct development patterns. In type 1, leukemic clones were derived from non-PNH clones in most cases, and the PNH phenotype of erythrocytes disappeared with progression. In one of our cases, however, the patient was diagnosed with concomitant PNH and AML-M6, and leukemic cells were observed alongside CD55-negative and CD59-negative PNH clones. In Type 2 cases such as this one, conversion of PNH is characterized by the coexistence of leukemic cells with PNH clones. Flow cytometry revealed that CD34-positive blast cells were deficient in CD55 and CD59. In Type 2, PNH clones do progress into malignancies, albeit rarely, demonstrating a distinct second development pattern of leukemic conversion from PNH.

## Introduction

Paroxysmal nocturnal hemoglobinuria (PNH) is a clonal hematopoietic stem cell disorder caused by a somatic mutation in phosphatidylinositol *N*-acetylglucosaminyltransferase subunit A (*PIGA*), whose gene product is required for the synthesis of glycosylphosphatidylinositol (GPI) anchors [1]. Patients with PNH have variable clinical courses. Most patients survive for decades after diagnosis of PNH, exhibiting chronic anemia, whereas a small number of patients with PNH have gone on to develop myelodysplastic syndrome (MDS)/acute myeloid leukemia (AML) after long periods. In the clinical course of PNH, the incidence of leukemic conversion from PNH has been reported to be 0.6–2.9% [2]. In most cases, PNH clones have diminished in the conversion to MDS/AML [3]. Cases of progression from PNH to AML were also reported, but clonal analysis was ambiguous in an era when flow cytometry techniques were not available. We have observed two cases: a case of MDS derived from PNH and a case of concomitant PNH with AML. Analysis of these two cases illustrates two distinct development patterns by which PNH may convert to MDS/AML.

## Case presentation

**Fig. 1:**
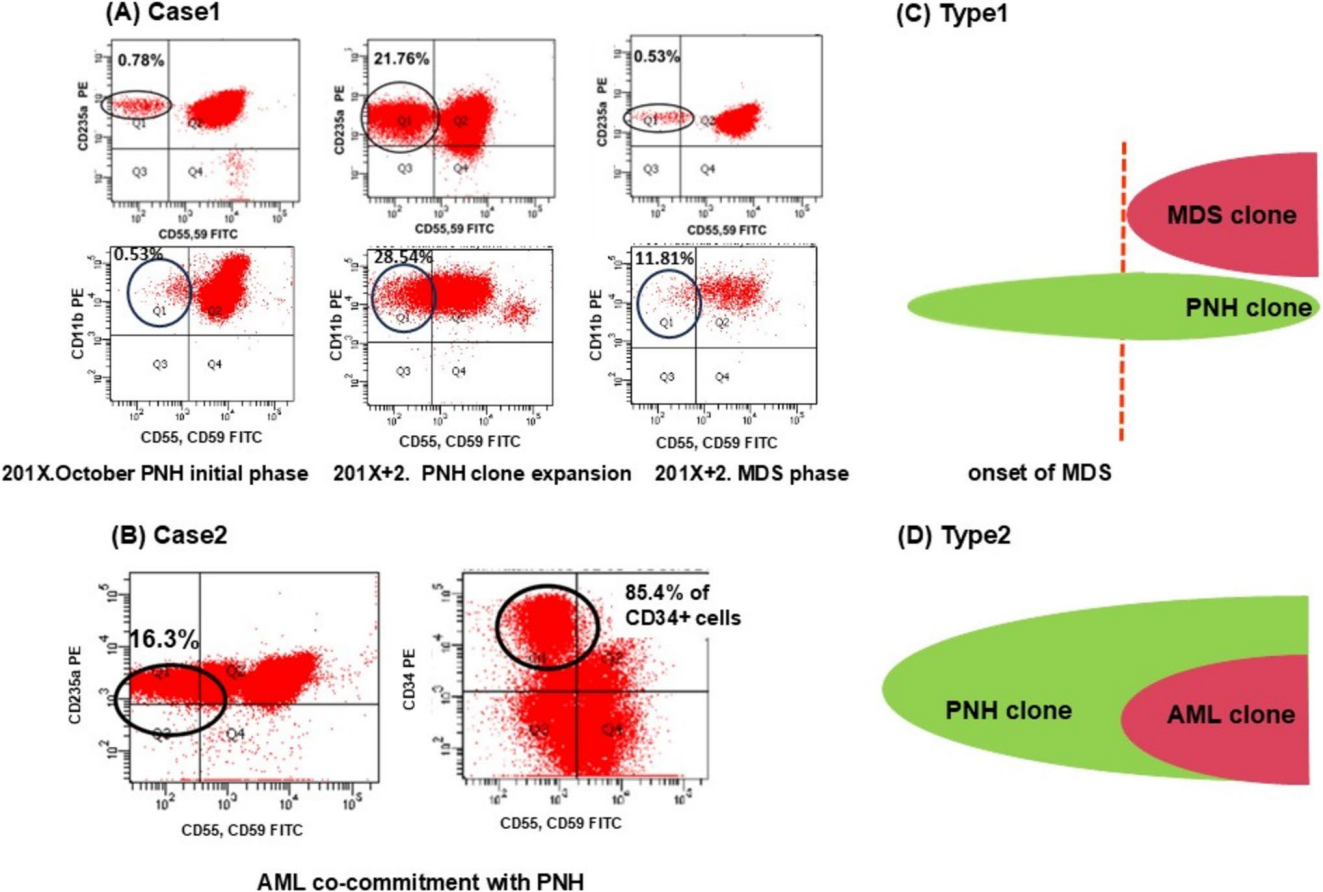
The populations of CD55(−) CD59(−) PNH clones changes in phases between the types of the development. Flow cytometry dot plot analyses of peripheral blood samples from patients. **A**, **C** PNH clone size was determined by measuring CD55(−) CD59(−) CD235a(+) erythroid cells and CD55(-) CD59(−) CD11b(+) neutrophil cells. PNH clone size had diminished over the development to MDS in case1. MDS cells were thought to be derived from non-PNH clone cells as shown in the scheme. **B**, **D** 16.3% of CD235a(+) erythroid cells and 85.4% of the CD34(+) blast cells consisted of CD55(−) CD59(−) PNH clones. Acute erythroid leukemia was concomitant with PNH and leukemic blast cells were thought to be derived from PNH clone cells. FITC, fluorescein isothiocyanate; PE, phycoerythrin

Case 1: A 42-year-old woman was observed with thrombocytopenia in a medical examination, which progressed to pancytopenia in 6 months. The bone marrow aspiration (BMA) yielded a marked hypocellular bone marrow, and flow cytometry (FCM) analysis performed on peripheral blood demonstrated CD55(−) CD59(−) granulocytes (approximately 2.3%) and red blood cells (approximately 0.6%). She was diagnosed with aplastic anemia stage 3 and received immunosuppressive therapy consisting of antithymocyte globulin (ATG), cyclosporine (CyA), and methylprednisolone (mPSL). Since then, she has been managed as an outpatient and received treatment with CyA and PSL. Her peripheral blood neutrophil and platelet count remain low (neutrophil: 0.5 × 10^9^–0.8 × 10^9^/L, hemoglobin: 60–80 g/L, platelet: 1.0–2 × 10^9^/L). Two years later, she was admitted to our hospital due to poorly controlled anemia. Her reticulocyte counts, unconjugated bilirubin levels, and lactate dehydrogenase (LDH) activity had increased and haptoglobin levels had decreased (hemoglobin: 56 g/L, reticulocyte: 163,500/μL, unconjugated bilirubin: 1.8 mg/dL, LDH: 1674 U/L). FCM analysis performed on peripheral blood revealed CD55(−) CD59(−) granulocytes (approximately 28.5%) and red blood cells (approximately 21.8%, Fig. 1A). She was remitted from hemolysis immediately with a dose escalation of PSL. However, her pancytopenia prolonged, and a BMA performed 2 months later revealed approximately 4.3% myeloblasts of nucleated cells, multinucleated erythroblasts, and megakaryocytes of multiple, widely separated nucleated cells. Cytogenetics studies showed monosomy 7 in 20 metaphases analyzed, and she was diagnosed with MDS-refractory cytopenia with multilineage dysplasia (RCMD). Notably, the proportion of CD55(−) CD59(−) granulocytes and red blood cells decreased when she was diagnosed with MDS (Fig. 1A). The patient underwent unrelated bone marrow transplant. After engraftment, she has been managed as an outpatient. Two months post-transplant, she was admitted to our hospital due to severe diarrhea. Colonoscopy revealed mucosal sloughing and diffuse mucosal defect in the whole small intestine. She was diagnosed with Stage 3 gastrointestinal graft versus host disease (GVHD). No significant skin rash or liver involvement was observed, and she was diagnosed with Grade III acute GVHD. She received immunosuppressive therapy consisting of mPSL and beclomethasone. A bone marrow biopsy did not show relapse of MDS. 295 days after transplantation, she died from acute GVHD as well as a bacterial infection which arose during immunosuppressive therapy.

Case 2: An 81-year-old man was observed with slight pancytopenia through an examination before surgery for lumbar spinal canal stenosis. His hemoglobin level was 129 g/L, white blood cell (WBC) count was 4.9 × 10^9^/L, and platelet count was 12.9 × 10^9^/L. General malaise and palpitations appeared from the next month, and he was referred to our hospital for further examination. His hemoglobin level was 81 g/L, WBC count was 2.1 × 10^9^/L with 12.0% blasts, and platelet count was 6.2 × 10^9^/L. BMA yielded a marked hypercellular bone marrow and erythroid dominance, representing approximately 47% myeloblasts of non-erythroid cells (NECs). Cytogenetic studies showed a complicated chromosome aberration: 45,X,-Y,−2,add(3)(p12),add(5)(q31),add(7)(q11.2),−9,add(15)(p11.2),+2mar in 12 of 20 metaphases analyzed. Furthermore, reticulocyte counts, unconjugated bilirubin levels, and LDH activity had increased and haptoglobin levels had decreased, suggesting intravascular hemolysis (reticulocyte: 89,800/μL, unconjugated bilirubin: 1.7 mg/dL, LDH: 1881 U/L). FCM analysis performed on peripheral blood demonstrated CD55(−) CD59(−) granulocytes (approximately 42%) and red blood cells (approximately 16%), consistent with paroxysmal nocturnal hemoglobinuria (PNH). Furthermore, 85.4% of the CD34-positive blast cells consisted of CD55- and CD59-negative PNH clones (Fig. 1B). The patient was diagnosed with concomitant PNH and acute erythroid leukemia. The patient received induction therapy with idarubicine (IDR) 12 mg/m^2^ 1 day and cytarabine (AraC) 100 mg/m^2^ daily for five consecutive days. At 4 weeks after induction therapy, blasts in both peripheral blood and bone marrow had increased and determined non-remission status. The patient died from leukemic progression to cerebral infarction as well as a bacterial pneumonia infection which arose during induction therapy.

## Discussion

The incidence of leukemic progression from PNH has been reported to be between 0.6 and 2.9%. In a literature review, 24 total cases of leukemic conversion from PNH were mentioned (Table 1) [1, 3–6]. In most of these cases (88%), PNH clones had diminished in the conversion of PNH to MDS/AML, which is thought to arise from the injured bone marrow as a second hematopoietic malignancy. However, the mechanisms of association of PNH with leukemic conversion remain unclear.

**Table 1:** Characteristics of patients in our cases and the literature

Case	sex	Age at diagnosis of PNH	PNH to diagnosis of leukemia/ MDS (month)	Type of leukemia/ MDS	Development patterns from PNH to AML / MDS	References
1 Current case 2	M	81	0	AML	Type 2	
2	M	81	1	ALL	Type 2	[1]
3	M	64	19	AML	Type 2	[3]
4	M	50	36	AML	Type 1	[4]
5	M	32	3	AML	Type 1	[4]
6	M	57	31	AML	Type 1	[4]
7		Child		AML	Type 1	[4]
8		Child		AML	Type 1	[4]
9	M	20	42	AML	Type 1	[4]
10			264	AML	Type 1	[4]
11	F	42	76	AML	Type 1	[4]
12	F	37	72	AML	Type 1	[4]
13	M	21	47	AML	Type 1	[4]
14	M	58	30	AML	Type 1	[4]
15	F	31	8	AML	Type 1	[4]
16	F	36	11	AML	Type 1	[4]
17	M	58	72	AML	Type 1	[4]
18	M	64	56	AML	Type 1	[4]
19	M	42	4	AML	Type 1	[4]
20	F	76	18	AML	Type 1	[4]
21	M	65	71	AML	Type 1	[4]
22				AML	Type 1	[4]
23			60	ALL	Type 1	[4]
24	F	21	60	ALL	Type 1	[5]
25	M	52	132	CML	Type 1	[6]
26 Current case 1	F	42	3	MDS	Type 1	

PNH, paroxysmal nocturnal hemoglobinuria; MDS, myelodysplastic syndromes; AML, acute myeloid leukemia; ALL, acute lymphoid leukemia; CML, chronic myeloid leukemia

The results of our studies and literature review reveal the existence of two types of leukemic conversion from PNH. In conversions of Type 1, leukemic blast cells were derived from non-PNH clone cells (Fig. 1C). As illustrated by Case 1 of our study, PNH clone size had diminished over the development to MDS. By contrast, conversions of Type 2 demonstrate leukemic cells coexisting with PNH clones (Fig. 1D). In Case 2 of our study, flow cytometry revealed CD34-positive blast cells were deficient in CD55 and CD59. These analyses indicated that leukemic blast cells were derived from PNH clone cells.

In conclusion, this report observed two patients who illustrated the two distinct development patterns from PNH to AML/MDS. PNH clones are typically considered to be benign clones which do not progress into malignancies. However, as illustrated by the possibility of Type 2 conversion, cases in which PNH clones do progress into malignancies, albeit rare, do exist, demonstrating a distinct second development pattern of leukemic conversion from PNH. Further research about PNH clone size during development of AML/MDS over the time is required to reveal the relationship between PNH and leukemic conversion.
